# Association mapping and genome prediction reveal a complex genetic architecture for resistance to black and frosty pod rot in cacao (*Theobroma cacao* L.) populations

**DOI:** 10.1002/tpg2.70322

**Published:** 2026-10-11

**Authors:** Elisa Jacquotte, James A. Holzwarth, Ana Zotta Mota, Mariela E. Leandro‐Muñoz, Bénédicte Rhoné, Evelyn T. Todd, Maud Lepelley, Laurence Bellanger, Patrick Descombes, Fabrizio Arigoni, Audrey Fillodeau, Eduardo Beche

**Affiliations:** ^1^ Société des Produits Nestlé SA, Nestlé Research Tours France; ^2^ Société des Produits Nestlé SA, Nestlé Research Lausanne Switzerland; ^3^ CATIE (Centro Agronómico Tropical de Investigacion y Enseñanza) Turrialba Cartago Costa Rica; ^4^ CIRAD, UMR AGAP Institut Montpellier France; ^5^ AGAP Institut, Université Montpellier, CIRAD, INRAE, Institut Agro Montpellier France

## Abstract

Cacao (*Theobroma cacao* L.) production is severely constrained by black pod rot (BPR) and frosty pod rot (FPR), two major diseases that cause substantial yield losses across the tropics. Breeding for disease resistance remains challenging due to long generation intervals and the complex genetic control of disease responses. Here, we used genome‐wide association studies (GWASs) and genomic prediction (GP) to dissect the genetic basis of BPR and FPR resistance in an interrelated multi‐parent F_1_ panel comprising 495 individuals derived from eight bi‐parental crosses. Whole‐genome sequencing of 495 F1 cacao plants yielded 103,479 high‐quality single‐nucleotide polymorphisms used for association mapping. GWAS identified 45 quantitative trait loci (QTLs) associated with BPR or FPR resistance, including a shared hotspot on chromosome 7 involved in both diseases. Disease resistance loci exhibited small individual effects (0%–8% variance explained), consistent with a highly polygenic architecture. Genomic prediction showed encouraging predictive abilities ranging from 0.36 to 0.64 for FPR and BPR. Multi‐trait GP models that incorporated secondary traits increased predictive ability between 1.3‐ and 2‐folds for frosty pod rot traits. Marker subset and training population analyses demonstrated that predictive ability plateaued at ∼1000 markers and ∼450 training individuals, respectively, with little improvements on accuracy after that. Our results highlight the polygenic nature of resistance to BPR and FPR and demonstrate the strong potential of incorporating secondary traits into a multi‐trait analyses for increasing accuracies for primary traits.

## Core Ideas

- Resistance to frosty pod rot and black pod rot in cacao is highly polygenic, with numerous small‑effect loci distributed across the genome.
- Genome‐wide association analyses revealed both additive and dominance effects for disease resistance traits, indicating that nonadditive genetic variation contributes to resistance.
- Genomic prediction achieved moderate to high predictive abilities for disease resistance and demonstrated the usefulness of prediction‐based methods for complex traits.
- Multi‐trait genomic prediction combined with secondary traits improved predictive abilities for frosty pod rot traits and number of pods.

## Plain Language Summary

Cacao production is affected by several diseases including black pod rot (BPR) and frosty pod rot (FPR), which cause significant yield losses in tropical regions. This study aimed to explore the genetic basis of resistance to BPR and FPR by using genomic analysis on a diverse panel of 495 cacao plants derived from eight parent crosses. We identified 45 genomic regions linked to resistance, with small individual effects indicating a complex genetic architecture. Genomic prediction methods showed better accuracy than traditional approaches, with accuracies ranging from 0.36 to 0.64 for disease‐related traits. Also, multivariate genomic prediction models increase accuracy compared to univariate models. The findings highlight the complex nature of resistance and the potential of genomic prediction to enhance breeding efficiency for disease‐resistant cacao.

**Abbreviations**
BLUE: best linear unbiased estimators
BPR: black pod rot
CV: cross‐validation
FPR: frosty pod rot
FPRe: frosty pod rot external lesion
FPRi: frosty pod rot internal lesion
GBLUP: genomic best linear unbiased predictor
GEBV: genomic estimated breeding value
GP: genomic prediction
MT‐GP: multi‐trait genomic prediction
ST‐GP: single‐trait genomic prediction
GWAS: genome‐wide association study
LD: linkage disequilibrium
LOOCV: leave‐one‐out cross‐validation
MAS: marker‐assisted selection
NOP: number of pods
NOHP: number of healthy pods
PA: predictive ability
QTL: quantitative trait locus
WB: witches’ broom

## 1 INTRODUCTION

Cacao (*Theobroma cacao L.)* is a tree native to the upper Amazonian region and today is cultivated across humid tropical regions worldwide. As a major export commodity, cacao supports the livelihoods of 6 million smallholder farmers, who account for approximately 95% of global production (Kozicka et al., 2018; Suh & Molua, 2022). Production is concentrated in West Africa, which contributes 56% of global output, with Côte d'Ivoire alone producing 36% of the total global production (1.9 million tons in 2024). South America and Asia follow with 21% and 13%, respectively (FAOSTAT, 2024).

Cacao production faces severe constraints from diseases, which cause 30%–40% yield losses globally (Bailey & Meinhardt, 2016). Three major fungal diseases are among the main culprits: frosty pod rot (FPR) caused by the fungi *Moniliophthora roreri*, witches’ broom (WB) caused by *Moniliophthora perniciosa*, and black pod rot (BPR) caused by different oomycetes from *Phytophthora* genus, including *Phytophthora capsici*, *Phytophthora tropicalis*, *Phytophthora citrophthora*, *Phytophthora megakarya*, and *Phytophthora palmivora*. Among these, *P. megakarya* is considered the most destructive and virulent (Marelli et al., 2019). In addition, the distribution of these diseases is not uniform across cacao‐producing regions. BPR is present in all producing countries and yet exhibits a prevalence for West Africa (Bailey & Meinhardt, 2016). FPR is present mostly in tropical America, and outbreaks have affected producing countries, leading to severe losses, such as a 72% decline in Costa Rican production between 1978 and 1983 (Bailey et al., 2018). Alarmingly, FPR continues to spread rapidly in newly affected areas like Bolivia (Phillips‐Mora et al., 2015), and its potential to spread into West Africa, where production relies on the highly susceptible Amelonado genotypes (Diaz‐Valderrama et al., 2020; Ploetz, 2006), poses a major threat to the chocolate industry.

Current diseases management relies on agricultural practices such as crop sanitation by removing infected pods, weeding, and spacing trees (Doungous et al., 2018; Soberanis et al., 1999). For BPR, using physical barriers can be beneficial as it reduces access by insect vectors (Rosmana et al., 2013). Copper‐based synthetic fungicides are reported to be efficient against BPR but present an elevated level of environmental toxicity (Ibrahim & Wahab, 2021). In addition, these solutions are still not cost‐effective. As a result, breeding genetically resistant material appears to be a promising long‐term solution for disease management. However, only a small fraction of the total cocoa genetic diversity has been used for disease‐resistance breeding in cacao (Zhang & Motilal, 2016), which increases the likelihood of diseases outbreaks.

In this context, quantitative trait loci (QTLs) studies have been conducted to identify specific linkage regions associated with resistance to BPR and FPR in cacao. Numerous QTL studies have reported the presence of resistance to black pod within cacao germplasm (Barreto et al., 2018; Brown et al., 2007; Clement et al., 2003; Crouzillat et al., 2000). Gutiérrez et al. (2021) identified 11 QTLs associated with FPR across six chromosomes and four QTLs associated with BPR across four chromosomes, showing the complex genetic mechanisms behind resistance against those diseases in cacao. The results of QTL mapping experiments can vary based on the population and the methods used for disease screening. Lanaud et al. (2009) performed a Meta‐QTL analysis across 16 QTL studies and identified chromosomes 2 and 8 as potential hotspots for BPR and FPR resistance. Overall, QTL studies are effective when a major gene is involved in resistance but often lack transferability across populations due to background effects and the genetic architecture of the trait.

To overcome these limitations, genome‐wide association studies (GWASs) and genomic prediction (GP) have emerged as powerful tools. GWAS relies on historical linkage disequilibrium (LD) to increase the power of association between phenotypic variation and single‐nucleotide polymorphisms (SNPs) within a population where structure is controlled. On the other hand, GP predicts progeny performance based on genomic estimated breeding values (GEBVs) using genome‐wide markers (Meuwissen et al., 2001). Practically, such approaches require to generate training populations (TPs) where genotypic and phenotypic markers are collected, training the statistical model in the dataset to subsequently test on the target population.

McElroy et al. (2018) used both methods across three cacao populations to assess WB and FPR resistance and observed that GP captured top performers better than GWAS, mainly because GP was able to capture the trait architecture better and markers associated with the diseases were not shared among all populations. Additionally, previous work on GP for cacao revealed promising results for traits of economic importance. Predictive ability for the average of beans weight was 0.59 (Ribeyre et al., 2017) and 0.61 for seed number (Bekele et al., 2022). Similar results were obtained by Romero Navarro et al. (2017) with prediction accuracy for FPR and BPR traits varying between 0.43 and 0.67, demonstrating the usefulness of GP to breed for disease resistance in cacao.

Cacao is a crop with many challenges for breeders. The long juvenile period of 3–5 years complicates the selection of fruit‐specific traits (Wickramasuriya & Dunwell, 2018) and delays selection based on phenotypic data. Also, cacao is an outbreeding species with a strong sexual incompatibility leading to populations that are mostly heterozygous (Lopes et al., 2011). Consequently, a cycle of selection can last up to a decade, taking at least 20 years to obtain an improved variety (Bekele & Phillips‐Mora, 2019). Cacao trials also require large land parcels and a high input of resources for maintenance and data acquisition. There is therefore an urgent need to develop genomic, and cost effective, approaches to accelerate genetic gain in cacao breeding.

Genomic tools are available for cacao breeding programs with microsatellites and SNPs (Kuhn et al., 2011; Santos et al., 2012), enabling to accelerate breeding processes. Recently, gene editing using CRISPR/Cas9 has also shown promising results in enhancing resistance to *P. tropicalis*, a causal strain of Black pod rot disease (Fister et al., 2018). However, incorporating traits of interest into high‐yielding germplasm remains time‐consuming. The objectives of this study were to identify genomic regions associated with resistance to BPR and FPR in eight bi‐parental cacao populations and to evaluate the potential of genomic prediction strategies for breeding disease‐resistant cacao varieties.

**Core Ideas**

- Resistance to frosty pod rot and black pod rot in cacao is highly polygenic, with numerous small‑effect loci distributed across the genome.
- Genome‐wide association analyses revealed both additive and dominance effects for disease resistance traits, indicating that nonadditive genetic variation contributes to resistance.
- Genomic prediction achieved moderate to high predictive abilities for disease resistance and demonstrated the usefulness of prediction‐based methods for complex traits.
- Multi‐trait genomic prediction combined with secondary traits improved predictive abilities for frosty pod rot traits and number of pods.

## 2 MATERIALS AND METHODS

### 2.1 Plant materials and experimental design

The study panel consisted of eight bi‐parental crosses generated at CATIE (Turrialba, Costa Rica). The six parental clones (CATIE‐R6, CHUAO‐120, EET‐95, PA‐51, ICS‐95, and EET‐399) were selected for their variable resistance response to FPR, BPR, and witches’ broom (WB), and quality attributes. For example, CATIE‐R6 is resistant to FPR, and on the other hand, PA‐51 is well known for a better response against BPR.

The crosses were interconnected by using CATIE‐R6 and ICS‐95 in four crosses each as hub‐parents. Each cross originally comprised 185 progenies, but the final dataset was reduced after mortality, phenotyping limitations, and genetic conformity filtering. The final number of trees used in this study comprised 495 F_1_ plants (Table 1).

**TABLE 1:** Crosses between CATIE parents and their target diseases.

Cross	Disease target	Number of trees
CATIE‐R6 × CHUAO‐120	FBR and WB	59
CATIE‐R6 × EET‐95	FPR	91
CATIE‐R6 × PA‐51	FPR and BPR	57
CHUAO‐120 × ICS‐95	WB and FPR	38
EET‐399 × CATIE‐R6	WB and FPR	81
EET‐399 × ICS‐95	WB and FPR	51
EET‐95 × ICS‐95	FPR	75
PA‐51 × ICS‐95	BPR and FPR	43

Abbreviations: BPR, black pod rot; FPR, frosty pod rot; WB, witches broom disease.

Trees were planted in a randomized complete block design at CATIE's La Lola experimental farm (10.0888° N, 83.3881° W; 40 m a.s.l.). The design included two sections, each containing four blocks of 185 trees (1480 trees). Resistance to FPR and BPR was assessed via artificial inoculations between 2016 and 2019, followed by field evaluations from 2020 to 2023.

### 2.2 Disease resistance screening

#### 2.2.1 Black pod rot

Artificial inoculations to evaluate the reaction against *P. palmivora* were performed using the paper‐disk method designed by Phillips‐Mora and Galindo (1989). Isolates of *P. palmivora* came from fruits naturally affected by BPR disease. Five‐month‐old, detached pods were inoculated with the zoospore suspension adjusted at a concentration of 160,000 spores per milliliter, absorbed on 0.5‐cm‐diameter filter paper discs, which were placed on two opposite sites in the middle of the pod. Fruits were covered with transparent plastic bags containing wet paper towels to maintain humidity and stored at room temperature. Disease severity was quantified by measuring the average diameter of the lesion 10 days after inoculation following the scale of Phillips‐Mora and Castillo (1999) and Leandro Muñoz et al. (2025a): 0.0–2.0 cm (highly resistant), 2.1–4.0 cm (resistant); 4.1–6.0 cm (moderately resistant), 6.1–8.0 cm (moderately susceptible), 8.1–10.0 cm (susceptible), and >10.0 cm (highly susceptible) (Figure S1). Since pod production is not uniform in cacao, multiple inoculation events happened for BPR between 2016 and 2019 to access the resistance profile of the genotypes. On average, each genotype was evaluated across 4.6 (SD 2.0) inoculation events and 9.0 (SD 4.3) pods on average across all events.

#### 2.2.2 Frosty pod rot

The artificial reaction to *M. roreri* was determined using the method of Sánchez et al. (1987) modified by Phillips‐Mora and Galindo (1988). Young pods (2–3 months old) were spray‐inoculated in the field with a spore suspension, adjusted at a concentration of 120,000 spores per milliliter, using a manual sprayer. Approximately 0.5 mL of the freshly prepared suspension was applied per pod. Fruits were then covered with a transparent plastic bag containing a paper towel and 50 mL of water to maintain humidity. Two days after inoculation, the plastic bags were opened to eliminate excess humidity and avoid water accumulation.

Disease severity ratings were measured both externally and internally nine weeks after inoculation. *Moniliasis* external severity (frosty pod rot external lesion [FPRe]) is based on the external appearance of each pod using a six‐point scale (Leandro Muñoz et al., 2025b; Sánchez et al., 1987): 0, healthy pod/no apparent symptoms; 1, oily dots; 2, humps (swellings); 3, premature yellowing and/or chocolate spot; 4, fungal mycelium appearance; and 5, abundant sporulation (Figure S2). *Moniliasis* internal severity (Frosty pod rot internal lesion [FPRi]) is based on the percentage of internal necrosis in each fruit when sectioned longitudinally using the following scale: 0, 0%; 1, 1%–20%; 2, 21%–40%; 3, 41%–60%; 4, 61%–80%; 5, >80% (Leandro Muñoz et al., 2025b; Sánchez et al., 1987). Internal and external severity uses the following scale to classify the response against the disease: 0.0–1.0 (highly resistant); 1.1–2.0 (resistant); 2.1–3.0 (moderately resistant); 3.1–4.0 (moderately susceptible); 4.1–5.0 (susceptible) (Figure S3). Multiple inoculation events happened for FPR evaluation between 2016 and 2019 to access the resistance profile of the genotypes. On average, each genotype was evaluated across 4.3 (SD 1.8) inoculation events and averaged 7.15 (SD 4.07) pods across all events.

### 2.3 Field data collection

In addition to the disease severity data collected by artificial inoculation, field data were collected from the F_1_ trees between 2019 and 2023. The traits collected were as follows: number of pods (NOP) as total number of pods produced per tree, and number of healthy pods (NOHP) as total number of pods that did not present any disease infection per tree. Natural Moniliasis incidence (relying on natural infection) was estimated (%) as MI $=\frac{\mathrm{Number}\;\mathrm{of}\;\mathrm{pods}\;\mathrm{with}\;\mathrm{Moniliasis}}{\mathrm{Number}\;\mathrm{of}\;\mathrm{pods}}\;\times \;100$, where number of pods with Moniliasis is the number of pods with frosty pod rot symptoms, and NOP is the total number of pods produced per tree.

### 2.4 Whole genome sequencing and variant calling

Total genomic DNA was extracted from 12 discs of fresh ground leaf tissues using the QIAGEN DNeasy 96 Plant Kit following the manufacturer's instructions. The protocol includes lysis followed by protein and polysaccharide elimination. The DNA is then purified using the selective binding properties of silica gel membranes. DNA integrity was evaluated using the Tape Station technology (Agilent) on 10% samples randomly selected.

We sequenced the genomes of 522 F_1_ progenies and the six parents used for the crosses (528 in total). The sequencing was performed using Illumina technology, on NovaSeq 6000 with paired‐end 150 bp reads. Libraries were prepared using the DNA‐Prep kit (Illumina) following manufacturer recommendations.

Demultiplexing and adapter trimming were performed using Illumina software. Read quality was assessed using FastQC (Andrews, 2010) with default parameters. Reads were aligned to the Criollo cocoa genome V2 reference (Argout et al., 2017) using bwa‐mem2 v2.1 (Li & Durbin, 2010). Mate coordinate and insert‐size information were added using samtools fixmate v1.17 (Danecek et al., 2021), and alignments were coordinate‐sorted using samtools sort. PCR and optical duplicates were marked and removed using samtools markdup (‐rs). Reads were further filtered using samtools view (‐F 3852 ‐q 25), excluding unmapped reads, reads with unmapped mates, secondary and supplementary alignments, QC‐failed reads, duplicate reads, and reads with a mapping quality (MQ) below 25.

#### 2.4.1 Variant calling and filtering

Variants were called jointly across all filtered BAM files using Graphtyper v2.7.7 (Eggertsson et al., 2017) processed in two segments per chromosome, with no prior VCF file used for the first‐pass calling. Resulting per‐segment VCFs were concatenated using bcftools concat (Danecek et al., 2021). Variant‐level filtering was applied using vcffilter (Müller et al., 2017) to retain sites with a heterozygous allele balance (ABHet) of 0 or greater than 0.33, a homozygous allele balance (ABHom) of 0 or greater than 0.97, a maximum alternate allele support ratio (MaxAASR) greater than 0.4, and an MQ greater than 30. Sites were further filtered using bcftools view to retain only those with an aggregated allele score (AAScore) greater than 0.5 and a Graphtyper “PASS” filter flag, restricting to biallelic SNPs (bcftools ‐m2 ‐M2 ‐v snps); multi‐allelic sites were excluded from downstream analysis. Genotypes were additionally required to have a minimum genotype quality (GQ) of 20 across all samples (bcftools view ‐i ‘MIN(FORMAT/GQ) > 20’) prior to phasing. Variants were further filtered to remove those with a minor allele frequency below 0.05, a Hardy–Weinberg Equilibrium *p*‐value below 0.0001, or a maximum missing genotype rate above 50%.

#### 2.4.2 Phasing and imputation

The remaining variants were phased using Beagle v5.4 (Browning et al., 2018) with the parameters “ne = 4 window = 100 burnin = 90 iterations = 5000 phase‐states = 30,” and genotypes with a posterior probability (GP) below 0.99 were treated as missing. The final filtered, phased VCF was used as a reference panel for imputation. Mapped reads were jointly re‐genotyped as described above using this phased VCF as a prior, and the resulting calls were again filtered using vcffilter and bcftools with the same criteria described above. Imputation was performed in two sequential passes: first with Beagle v4 (gprobs = true), followed by Beagle v5.4 (ne = 500, window = 800, burnin = 90, iterations = 5000, phase‐states = 500; gp = true). After each imputation pass, genotypes with a posterior probability (GP) below 0.99 were treated as missing (bcftools view ‐i “FORMAT/GP ≥ 0.99”).

#### 2.4.3 Final variant filtering

Variants were first filtered using PLINK v1.90b6.18 (–geno 0.2 –maf 0.05) to exclude variants with a missing call rate greater than 20% and a minor allele frequency below 0.05. Variants in linkage disequilibrium were then removed from this filtered set using PLINK “–allow‐extra‐chr –indep‐pairwise 100 5 0.5” (a sliding window of 100 variants, shifted by five variants at each step, pruning variants with a pairwise *r* ^2^ greater than 0.5). Only SNPs assigned to the 10 *T. cacao* chromosomes were retained for further analysis. After these additional filters and LD pruning, a set of 103,479 high‐quality variants was retained. Finally, 495 genotypes were retained for further analysis after filtering for conformity within each cross.

### 2.5 Data analysis

#### 2.5.1 Phenotypic data

In the phenotypic data, diseases‐related traits (BPR, and FPR internal and external) were analyzed using the following mixed linear model in ASReml‐R (Butler et al., 2023):$\;y_{ij}=\mu +\beta_{i}+I_{j}+\beta I_{ij}+\varepsilon_{ij}$, where $y_{ij}$ is the observed phenotypic value for genotype *i* evaluated in the inoculation event *j*; μ is the overall mean; $\beta_{i}$ is the fixed effect of genotype *i*; $I_{j}$ is the random effect of the inoculation event *j*; $\beta I_{ij}$ is the random interaction effect of genotype *i* and inoculation event *j*; and $\varepsilon_{ij}$ is the residuals. Residual errors were assumed to be independent and identically distributed as $\varepsilon_{ij}∼\;N\left(0,\sigma_{e}^{2}\right)$.

For field‐related traits, the following model was used: $y_{ijklm}=\mu +\beta_{i}+\gamma_{j}+B_{k(j)}+S_{lm(j)}+\varepsilon_{ijklm}$, where $y_{ijklm}$ is the observed value for genotype *i* for year *j*, located at block *k*, row *l*, and column *m*; μ is the overall mean; $\beta_{i\;}\;\mathrm{is}\;$ the fixed effect of genotype *i*; $\gamma_{j}$ is the random effect of year *j*; $B_{kj}$ is the random effect of block *k* nested within year *j*; $S_{lm(j)}$ is the spatial effect for row *l* and column *m* within year *j*, modeled using a first‐order autoregressive process in both row and column directions [AR1(R) × AR1(C)]; and $\varepsilon_{ijklm}$ is the residual error. Year‐specific residual variances were modeled using a direct‐sum structure, assuming independent and identically distributed residuals within each year: $\varepsilon_{ijklm}∼\;N\left(0,\sigma_{e,j}^{2}\right)$.

Next, for all the traits evaluated, adjusted means were extracted for each genotype and used to perform the marker‐trait association and genomic prediction analysis.

#### 2.5.2 Genome‐wide association analysis

GWAS analyses were performed using the GWASpoly package in R (Rosyara et al., 2016). This package employs a Q (PCs) + random polygenic effect to control population structure, similar to the K model for GWAS (Yu et al., 2006). To avoid the double fitting of the candidate markers both as a random effect on the genetic relationship matrix and as a fixed effect for the evaluation of the association with the phenotype and for computational efficiency, we used the leave‐one‐chromosome‐out method (Yang et al., 2014). It consists of calculating a different covariance matrix for each chromosome based on the markers from all the other chromosomes.

The markers were next tested for significance with an additive and simplex dominance model. For the additive model, genotype data were encoded as 0 for the homozygous major allele, 1 for the heterozygous, and 2 for the homozygous alternative allele. For the simplex dominance model, both homozygous major and minor alleles were encoded as 0 and the heterozygous as 1. For a diploid, the simplex dominance model represents complete dominance. For 1‐dom‐ref, 0/0 and 0/1 genotypes are treated as equivalent, while for 1‐dom‐alt, the 0/1 and 1/1 genotypes are equivalent. Quantile‐plots were also produced to overview any bias in the GWAS model.

For the GWAS analysis, an SNP matrix of 103,479 high‐quality markers was used. The *p* value threshold for multiple tests was set using M.eff function implemented in the GWASpoly package. This test uses the LD between markers to estimate an effective number of markers, reducing the number of tests. For each QTL, the most significant marker within 6 Mb was returned using the get.QTL function. To identify significant QTLs in a multiple QTL model, the function fit.QTL was used to determine the *p* value and percentage of explained phenotypic variability by the QTL (*R* ^2^). Instead of testing each QTL one by one fit.QTL fits all QTLs in one model based on a backward elimination (removes least significant QTLs).

Population structure was assessed using principal component analysis (PCA) based on the SNP marker dataset, using the function snp.pca from the R package ASRgenomics (Gezan et al., 2022). PCA was performed to visualize genetic diversity and identify potential population stratification among individuals. To quantify realized genomic relationships among individuals, an additive genomic relationship matrix (GRM) was constructed using the VanRaden method (VanRaden, 2008), implemented in the R package AGHmatrix (Amadeu et al., 2023).

#### 2.5.3 Genomic predictions

Three genomic best linear unbiased predictor (GBLUP) prediction models were fitted using the ASReml‐R package (Butler et al., 2023). In all genomic prediction models, the response variable was the genotype‐adjusted means (adjusted best linear unbiased estimators [BLUE] values). The first one was an additive genetic mixed model (GBLUP_A_) as follows: $y=X\beta +Z_{a}a+\varepsilon ,$ where y is the vector of adjusted phenotypic observations, β is the vector of fixed effects, represented here by the overall mean, and ε is the vector of residual effects, assumed to follow: $\varepsilon \;∼\;N(0,I\sigma_{e}^{2})$. The term a represents the vector of random additive genomic effects, assumed to follow: $a\;∼\;N(0,G_{\mathrm{add}}\sigma_{a}^{2})$, where $G_{\mathrm{add}}$ is the additive GRM and $\sigma_{a}^{2}$ is the additive genetic variance. The matrix X is the incidence matrix relating fixed effects to phenotypic observations, and $Z_{a}$ is the incidence matrix relating individual additive genomic effects to observations. The additive GRM $G_{\mathrm{add}}$ was constructed following the method of VanRaden (2008) in the AGHmatrix R package (Amadeu et al., 2023).

The second model was an additive + dominance genetic mixed model (GBLUP_AD_) fitted as follows: $y=X\beta +Z_{a}a+Z_{d}d+\varepsilon$, where d is the vector of random dominance genomic effects, assumed to follow $d\;∼\;N(0,G_{\mathrm{dom}}\sigma_{d}^{2})$, where $G_{\mathrm{dom}}$ is the dominance GRM and $\sigma_{d}^{2}$ is the dominance genetic variance, and $Z_{d}$ is the incidence matrix relating individual dominance genomic effects to observations. Dominance GRM $G_{\mathrm{dom}}$ was constructed following Vitezica et al. (2013) in the AGHmatrix R package (Amadeu et al., 2023).

Genomic heritability ($h_{g}^{2}$) was calculated for the additive and additive + dominance GBLUP model (de Los Campos et al., 2015) as follows: $h_{A}^{2}=\frac{\sigma_{a}^{2}}{\sigma_{a}^{2}+\sigma_{e}^{2}}$, where $\sigma_{a}^{2}$ is the additive genetic variance calculated from the additive **G** matrix, and $\sigma_{e}^{2}$ is the residual variance, and $h_{\mathrm{AD}}^{2}=\frac{\sigma_{a}^{2}+\sigma_{d}^{2}}{\sigma_{a}^{2}+\sigma_{d}^{2}+\sigma_{e}^{2}},$ where $\sigma_{a}^{2}$ is the additive genetic variance calculated from the additive **G** matrix, $\sigma_{d}^{2}$ is the dominance genetic variance calculated from the dominance **G** matrix and $\sigma_{e}^{2}$ is the residual variance.

The third genomic prediction model was a multi‐trait genomic prediction (MT‐GP) model, fitted as follows: y=μ+Zg+ε, where y is the vector of adjusted means for the traits (n×t; n genotypes and t traits), μ is the vector of trait means, Z is the incidence matrix relating observations to individuals, g is the vector of genetic effects of the individuals, and ε is the vector of residuals. The genetic effects were assumed to follow a multivariate normal distribution, g∼MVN(0,∑⊗ G), where G is the additive GRM constructed by the method of VanRaden (2008), ∑ is the genetic variance‐covariance matrix among traits fitted using a heterogeneous correlation structure, and ⊗ denotes the Kronecker product of the two matrices. Residuals were assumed to follow ε∼MVN(0,R⊗ I), where R is the unstructured residual variance‐covariance matrix between traits estimated using a heterogeneous correlation structure, I is the identity matrix, and MVN denotes the multivariate normal distribution. The MT‐GP models were compared to the standalone GBLUP_A_ (single‐trait genomic prediction [ST‐GP]). For all the genomic prediction models described above, an LD‐trimmed SNP matrix (24,218 SNPs) was used to run the analysis.

The influence of marker density on genomic prediction performance was investigated by fitting the additive GBLUP model (GBLUP_A_) using the rrBLUP package (Endelman, 2011) in 13 randomly sampled SNP subsets containing 50, 100, 250, 500, 1000, 2.5k, 5k, 10k, 20k, 40k, 60k, 80k, and 100k markers. The marker subsets were balanced between chromosomes, and the process was repeated 20 times with a fivefold cross‐validation (5‐Fold CV); in each iteration, a new additive relationship matrix was computed.

To evaluate the effect of TP size, the same model from marker density was fitted using training sets ranging from 10 to 460 individuals, with increments of 20 individuals between successive scenarios. The process was repeated 100 times, and each training set contained genotypes from all families. For marker density and TP size, we used the full SNP matrix (103,479 SNPs) as the genotypic matrix.

#### 2.5.4 Cross‐validation schemes and predictive ability

Two cross‐validation schemes were tested: The first one was a 5‐Fold CV scheme repeated 100 times for each model across all the families. Genotypes were randomly assigned to five approximately equal subsets (folds). In each iteration, four folds were used as the training set and the remaining fold was used as the validation set. The second cross‐validation method was a leave‐one‐out cross‐validation (LOOCV) scheme to assess across‐populations predictive ability. The dataset consists of eight families (crosses), and in each iteration, one population was excluded from the training set and used as the validation population, while the remaining seven populations were used to train the model. This process was repeated eight times so that each population served once as the validation set.

For all the genomic prediction models and cross‐validation schemes, the predictive ability was calculated as the Pearson correlation between adjusted means (adjusted BLUE) and GEBVs. To calculate the predictive ability for marker‐assisted selection (MAS) in each trait, we selected significant additive markers identified in the GWAS analysis and fit a regression model using the *lm* function from the lme4 package (Bates et al., 2015), considering markers as fixed effects.

#### 2.5.5 Phenotypic and genotypic correlation

Phenotypic correlations among traits were assessed using Spearman's rank correlation coefficient (*r*), which measures monotonic relationships between variables without assuming normality. Correlation coefficients were calculated using pairwise complete observations, and statistical significance was evaluated using Spearman's rank correlation test. Significant correlations (*p* < 0.05) were visualized using a correlation matrix.

The genetic correlation among the traits was calculated using the unstructured genetic‐covariance matrices fitting a multi‐trait model (MT‐GS), as described in the third genomic selection model (Section 2.5.3) using ASReml‐R package (Butler et al., 2023).

## 3 RESULTS

### 3.1 Phenotypic variation and population structure

A PCA was performed to explore the genetic structure among individuals using the genetic relationship matrix. The first two principal components (PC1 and PC2) accounted respectively for 15.5% and 9.2% of the total genetic variation (Figure 1A). The analysis revealed clear genetic differentiation among the eight bi‐parental families. Families derived from EET 399 showed the greatest separation from the remaining crosses, consistent with their distinct pedigree backgrounds. Similar results were identified in the GRM (Figure 1B). The matrix displays the self‐relatedness on the diagonal and relatedness between individuals on the off diagonal with each family color coded. It was observed that there is a higher relationship between families of half‐sibs and related genotypes. For example, CHUAO‐120 and ISC‐95 are both Trinitario accessions (mix between Criollo and Amelonado groups), which explain their genetic proximity.

**FIGURE 1:**
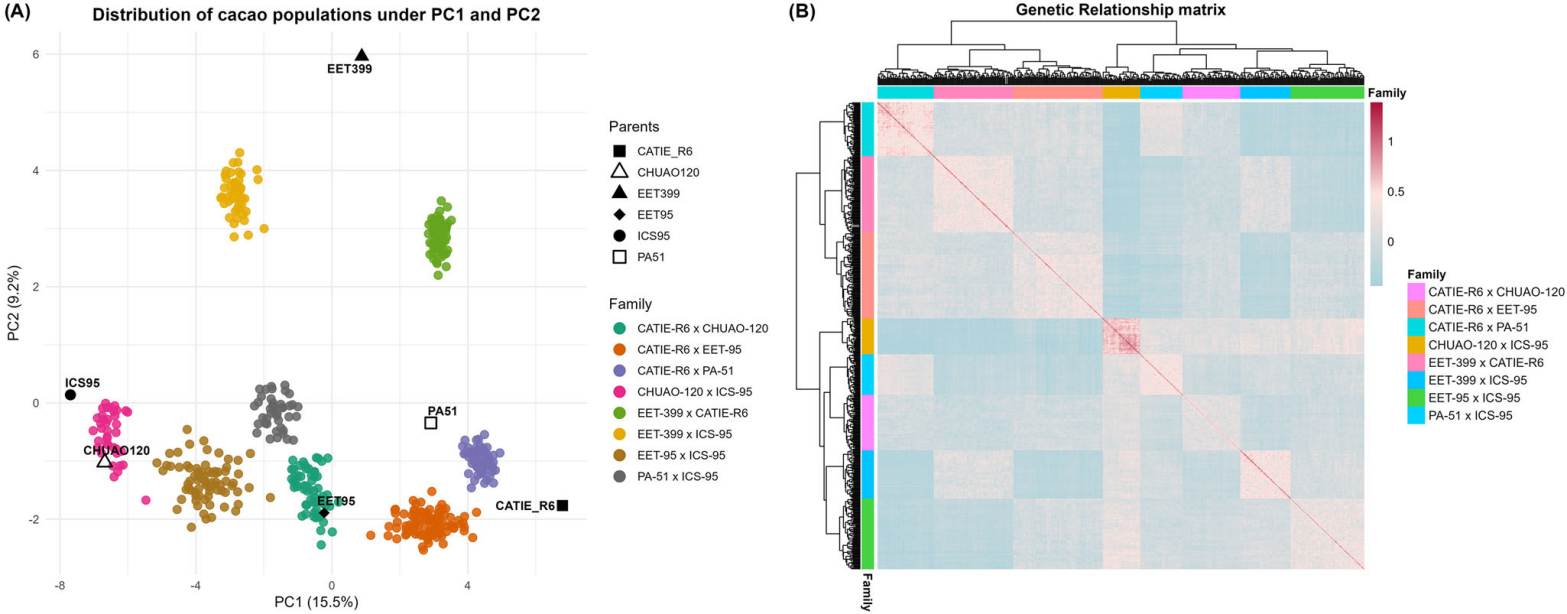
Distribution of the F_1_ families from eight cacao crosses along PCA axes 1 and 2. Each color represents one cross, and each parent is represented by different symbols in black color (A). Genomic relationship matrix (B).

All traits evaluated displayed a unimodal skewed distribution, except for FPRe and NOP that have a more normal distribution (Figure 2). FPRi and FPRe showed a mean of 3.1 and 2.6, respectively, suggesting that most individuals have moderate resistance to FPR (Table S1). Only six genotypes were characterized as highly resistant with a score of 1 for FPRe or FPRi. Regarding BPR, the panel average was 23.3 cm lesion size with a range depending on the population from 0.8 to 34 cm, placing most of the population as susceptible to BPR. Only seven individuals were found resistant, based on lesions size inferior or equal to 4 cm. Regarding hybrids that showed resistance to FPR and BPR only three were classified as resistant to both diseases (FPRe and FPRi = 1.1–2.0 scale and BPR = 2.1–4.0 cm lesion size).

**FIGURE 2:**
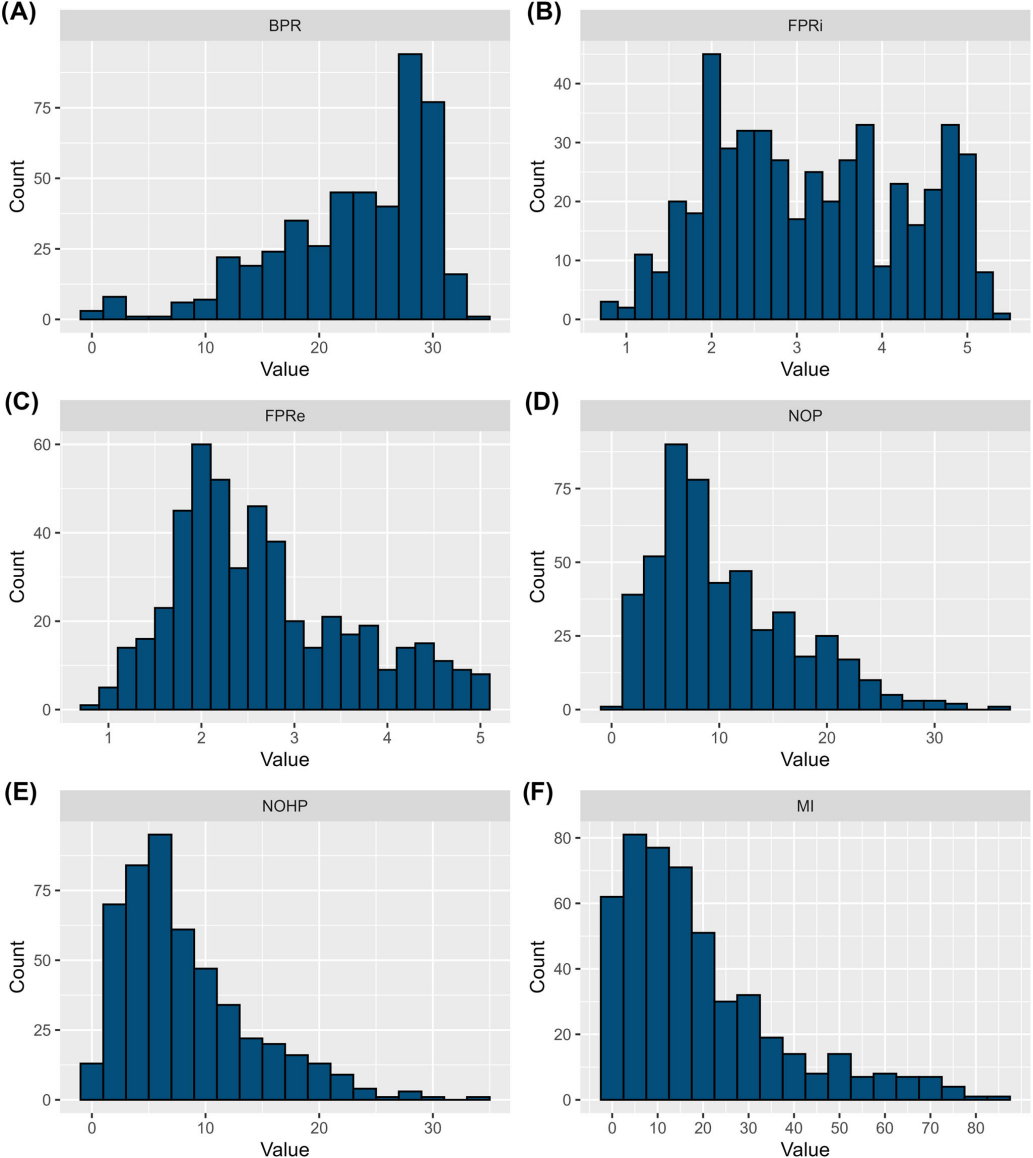
Phenotypic distribution of six cacao traits: (A) black pod rot (BPR), (B) frosty pod rot internal lesion (FPRi), (C) frosty pod rot external lesion (FPRe), (D) number of pods (NOP), (E) number of healthy pods (NOHP), and (F) Moniliasis incidence (MI).

For the traits evaluated under fields conditions, NOP, NOHP, and MI, averages are 10.3 pods, 8.1 pods, and 19.7%, respectively. Most cacao trees are moderate to low‐yield performers and are clearly exposed to FPR disease under field conditions. Correlations between traits (Figure S4), confirmed strong concordance between FPRi and FPRe symptoms (*r* = 0.93, *p* < 0.05), and moderate relationships between MI and FPRe (*r* = 0.58, *p* > 0.05) and FPRi (*r* = 0.53, *p* > 0.05), demonstrating a good level of concordance between artificial inoculation and field evaluation for FPR. However, it is important to highlight that there is a difference between artificial inoculation and natural incidence of FPR, since natural incidence is very dependable of environmental conditions (temperature and humidity) for dispersion (spores) and infection. In that scenario, artificial inoculation provides a more accurate scale of resistance between genotypes. In contrast, no significant correlation was observed between BPR and FPR traits.

Genomic correlations (Figure S5) showed a very similar pattern highlighting the high genetic correlation between FPRi and FPRe (0.98, SE = 0.007), and among MI and FPRe (0.93, SE = 0.04), and FPRi (0.92, SE = 0.04). Also, the results showed low genetic correlation between BPR and FPRi (0.06, SE = 0.15), and FPRe (0.13, SE = 0.15), demonstrating that these traits are largely genetically independent.

### 3.2 Genome‐wide association analysis

#### 3.2.1 Additive model

We first ran an additive model to identify loci with additive effects on the traits. Quantile–quantile (QQ) plots suggest that kinship relationships (*K*) and population structure (*Q*) were controlled in the GWAS additive and dominance model for all traits (Figure S6). Overall, we identified 16 significant QTLs across five traits for the additive model (Figure 3, Figure 4, and Table 2). For diseases‐related traits including BPR, FPRe, FPRi, and MI, 14 regions were identified in chromosomes 1, 2, 3, 4, 7, 9, and 10 (Figure 3 and Table 2). A hotspot for BPR and FPR was identified on chromosome 7 explaining between 2% and 4% of the phenotypic variation. Regarding BPR, an additional significant genomic region was detected on chromosome 10; however, this QTL was not significant when tested in association with the QTL on chromosome 7 (Table 2). For FPR traits, four regions were associated with FPR internal and external, explaining between 1% and 5% of the variation (Table 2). For FPRi one additional QTL was identified on chromosome 9, in the same region a significant QTL was identified for MI. The number of regions significantly associated with BPR and FPR reveals a complex genetic architecture for the traits, especially for FPR where six regions across the genome were identified.

**FIGURE 3:**
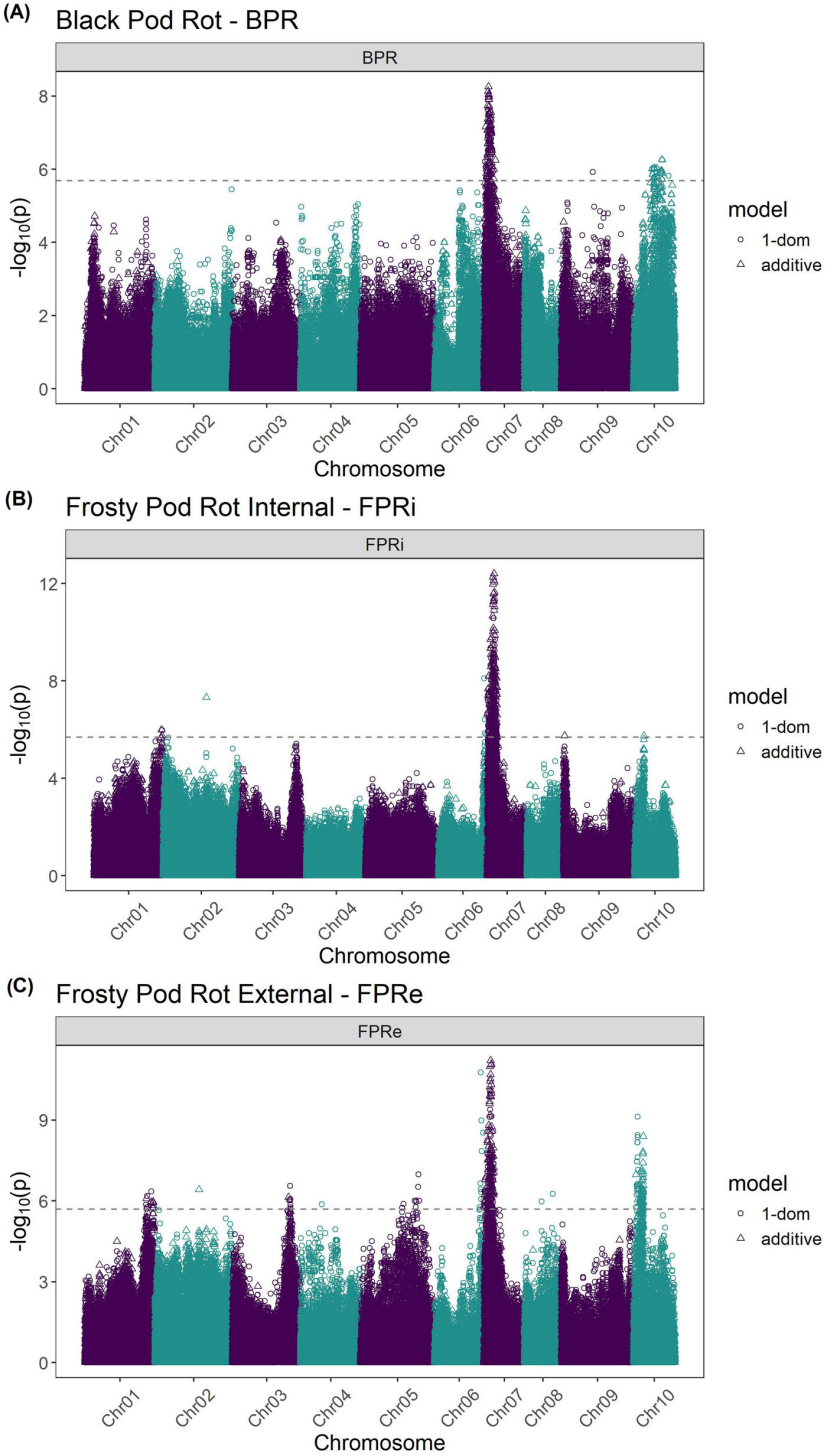
Manhattan plot of the additive and dominance genome‐wide association models for diseases‐related traits: (A) black pod rot (BPR), (B) frosty pod rot internal lesion (FPRi), and (C) frosty pod rot external lesion (FPRe). The dotted horizontal line corresponds to the Meff significance threshold of −log10 (P) = 5.69.

**FIGURE 4:**
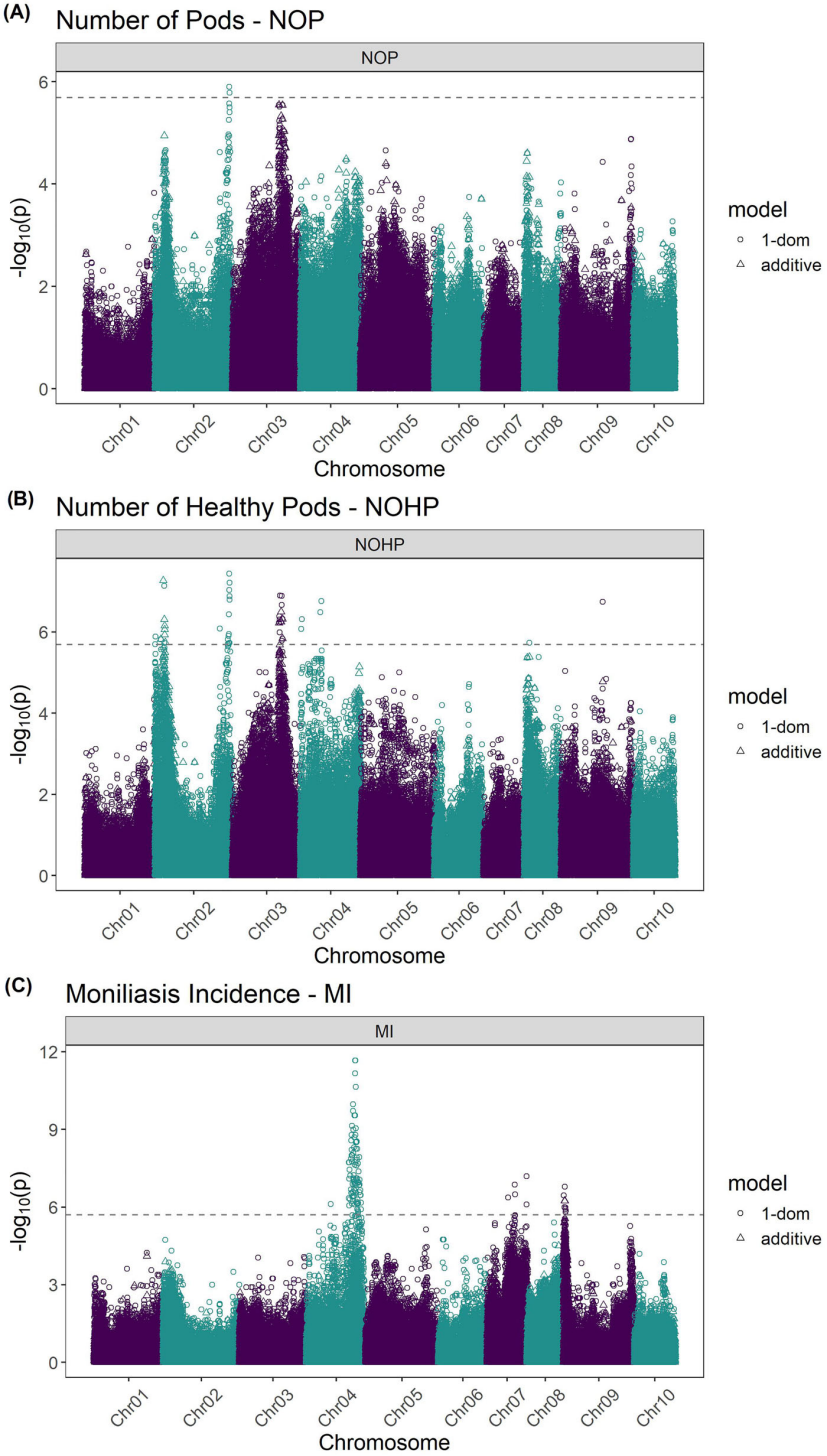
Manhattan plot of the additive and dominance genome‐wide association for yield‐related traits: (A) number of pods (NOP), (B) number of healthy pods (NOHP), and (C) Moniliasis incidence (MI). The dotted horizontal line corresponds to the Meff significance threshold of −log10 (P) = 5.69.

**TABLE 2:** Summary of significant quantitative trait loci (QTLs) associated with diseases resistance and yield‐related traits along with explained variation (*R* ^2^) by multiple QTL model (fit.QTL) for additive and dominance models.

Trait	Model	Marker	Chrom	Position	LOD	Effect	Fit.QTL model	
							*p* value	*R* ^2^
BPR	additive	chr7:2433336:SG	Chr07	2433336	8.26	2.5	0.000***	0.02
	additive	chr10:15002837:SG	Chr10	15002837	6.26	−4.7	1.000	0.00
	1‐dom‐alt	chr7:1981723:SG	Chr07	1981723	7.63	3.6	0.013**	0.01
	1‐dom‐alt	chr10:15002837:SG	Chr10	15002837	6.26	−4.7	1 ns	0.00
	1‐dom‐ref	chr9:16801647:SG	Chr09	16801647	5.93	5.8	0***	0.03
	1‐dom‐ref	chr10:18524168:SG	Chr10	18524168	5.82	4.4	0.335 ns	0.00
FPRi	additive	chr1:36239636:SG	Chr01	36239636	5.99	−0.6	0.478 ns	0.00
	additive	chr2:23293750:SG	Chr02	23293750	7.32	−0.3	0.000***	0.05
	additive	chr7:3640325:SG	Chr07	3640325	12.41	−0.9	0.000***	0.04
	additive	chr9:546283:SG	Chr09	546283	5.75	−0.4	0.000***	0.04
	additive	chr10:4942989:SG	Chr10	4942989	5.73	0.4	0.000***	0.03
	1‐dom‐alt	chr7:3207582:SG	Chr07	3207582	10.58	−0.9	0.210 ns	0.00
	1‐dom‐ref	chr1:36570206:SG	Chr01	36570206	5.97	−0.6	0.030*	0.01
	1‐dom‐ref	chr2:2682013:SG	Chr02	2682013	5.67	0.5	0.000***	0.03
	1‐dom‐ref	chr6:24617750:SG	Chr06	24617750	8.1	0.9	0.000***	0.08
	1‐dom‐ref	chr7:4090067:SG	Chr07	4090067	6.26	0.6	0.256 ns	0.00
FPRe	additive	chr1:32698758:SG	Chr01	32698758	6.08	−0.5	0.608 ns	0.00
	additive	chr2:23293750:SG	Chr02	23293750	6.42	−0.3	0.000***	0.04
	additive	chr3:29796616:SG	Chr03	29796616	6.14	0.4	0.000***	0.02
	additive	chr7:3414950:SG	Chr07	3414950	11.21	−0.7	0.000***	0.03
	additive	chr10:4942989:SG	Chr10	4942989	8.4	0.4	0.131 ns	0.01
	1‐dom‐alt	chr1:32776142:SG	Chr01	32776142	5.71	−0.6	0.000***	0.04
	1‐dom‐alt	chr3:30689395:SG	Chr03	30689395	6.56	0.4	0.124 ns	0.01
	1‐dom‐alt	chr4:11124742:SG	Chr04	11124742	5.88	0.6	0.910 ns	0.00
	1‐dom‐alt	chr5:22769639:SG	Chr05	22769639	5.89	0.5	0.168 ns	0.00
	1‐dom‐alt	chr5:30738896:SG	Chr05	30738896	6.98	0.6	0.070 ns	0.01
	1‐dom‐alt	chr7:3207582:SG	Chr07	3207582	10.11	−0.7	0.040*	0.01
	1‐dom‐alt	chr8:9139851:SG	Chr08	9139851	5.98	0.9	0.262 ns	0.00
	1‐dom‐alt	chr10:2052605:SG	Chr10	2052605	9.13	0.7	0.000***	0.02
	1‐dom‐ref	chr1:35249093:SG	Chr01	35249093	6.36	−0.6	0.971 ns	0.00
	1‐dom‐ref	chr2:2007131:SG	Chr02	2007131	5.66	0.4	0.000***	0.03
	1‐dom‐ref	chr3:30810112:SG	Chr03	30810112	5.86	−0.6	0.221 ns	0.00
	1‐dom‐ref	chr5:28764400:SG	Chr05	28764400	6	−0.5	0.011**	0.01
	1‐dom‐ref	chr6:24617750:SG	Chr06	24617750	10.77	0.8	0.000 ns	0.06
	1‐dom‐ref	chr7:8059425:SG	Chr07	8059425	5.67	−0.5	0.177 ns	0.00
	1‐dom‐ref	chr8:15106895:SG	Chr08	15106895	6.27	−1.0	0.703 ns	0.00
	1‐dom‐ref	chr10:4942989:SG	Chr10	4942989	6.8	0.4	0.850 ns	0.00
NOP	1‐dom‐ref	chr2:39434827:SG	Chr02	39434827	5.9	−3.9	0.000***	0.04
NOHP	additive	chr2:4212106:SG	Chr02	4212106	7.27	3.0	0.000***	0.04
	additive	chr3:25845694:SG	Chr03	25845694	6.49	3.0	0.000***	0.03
	1‐dom‐alt	chr2:4908325:SG	Chr02	4908325	7.14	3.0	0.029*	0.01
	1‐dom‐alt	chr3:25277674:SG	Chr03	25277674	6.9	3.4	0.822 ns	0.00
	1‐dom‐alt	chr8:2683156:SG	Chr08	2683156	5.73	2.9	0.000***	0.02
	1‐dom‐ref	chr2:258033:SG	Chr02	258033	5.88	−3.1	0.013**	0.01
	1‐dom‐ref	chr2:39434827:SG	Chr02	39434827	7.44	−3.8	0.000***	0.03
	1‐dom‐ref	chr4:705802:SG	Chr04	705802	6.31	−3.9	0.65 ns	0.00
	1‐dom‐ref	chr4:11078964:SG	Chr04	11078964	6.76	−4.2	0.550 ns	0.00
	1‐dom‐ref	chr9:22105881:SG	Chr09	22105881	6.74	−4.1	0.131 ns	0.01
MI	additive	chr4:27155942:SG	Chr04	27155942	5.71	−8.2	0.093 ns	0.01
	additive	chr9:561160:SG	Chr09	561160	6.24	−10.6	0.005*	0.02
	1‐dom‐alt	chr4:12941012:SG	Chr04	12941012	6.11	−12.3	0.006 ns	0.02
	1‐dom‐alt	chr4:24550119:SG	Chr04	24550119	9.13	−13.2	0.258 ns	0.00
	1‐dom‐alt	chr9:897202:SG	Chr09	897202	6.78	8.3	0.000***	0.02
	1‐dom‐ref	chr4:26141483:SG	Chr04	26141483	11.67	22.2	0.000***	0.05
	1‐dom‐ref	chr7:15002447:SG	Chr07	15002447	6.86	11.8	0.265 ns	0.00
	1‐dom‐ref	chr7:21241950:SG	Chr07	21241950	7.19	12.4	0.130 ns	0.01

Abbreviations: BPR, black pod rot; FPRe, frosty pod rot external lesion; FPRi, frosty pod rot internal lesion; NOP, number of pods; NOHP, number of healthy pods; ns, not significant; MI, Moniliasis incidence.

***, **, and * denote significance of 0.000, 0.01, and 0.05.

Regarding yield‐related traits, two regions were associated with NOHP on chromosomes 2 and 3 accounting for 3%–4% of the observed phenotypic variation (Figure 4 and Table 2). Regarding the trait NOP no significant markers were identified (Figure 4A).

#### 3.2.2 Dominance model

Under the dominance model, we identified 40 regions associated with six traits across the genome, more than double compared with the additive model (Figures 3 and 4). For BPR and FPR, 31 significant regions were identified, explaining between 0% and 8% of the phenotypic variation across all 10 chromosomes. Similar phenotypic variation was identified in the additive model (Table 2). Five regions on chromosomes 1, 2, 6, 7, and 9 were found to overlap between FPRe and FPRi. The same hotspot region on chromosome 7 was identified on the dominance model for FPR and BPR explaining very little of (0%–1%) of the phenotypic variance.

For NOP, one region was identified on chromosome 2, and for NOHP, eight regions were significant on chromosomes 2, 3, 4, 8, and 9. For MI, six regions were associated with the trait on chromosomes 4, 7, and 9. Comparing significant regions identified in the additive and dominance models, several regions overlap between the models for different traits, suggesting additive and dominance effects are not totally independent in the populations and traits studied.

Trait heritability has been estimated with genomic additive ($h_{A}^{2}$) and genomic additive + dominance ($h_{\mathrm{AD}}^{2}$) models (Table 3). $h_{A}^{2}$ varied from 0.32 to 0.69 and $h_{\mathrm{AD}}^{2}$ varied from 0.33 to 0.75. BPR, FPRe, and FPRi showed high values of $h_{A}^{2}$ (0.61–0.69) and $h_{\mathrm{AD}}^{2}$ (0.63–0.75). The moderate to high $h_{A}^{2}$ estimates suggest that additive genetic variance accounts for a significant amount of the total genetic variance for the traits studied. However, we also observed an improvement in heritability measurements when the dominance component was added in the model ($h_{\mathrm{AD}}^{2}$).

**TABLE 3:** Predictive ability of the genomic prediction models (GBLUP_A_ and GBLUP_AD_) across six traits in cacao.

Trait	MAS			GBLUP_A_		GBLUP_AD_		Trait average
				5‐fold CV	LOOCV	5‐fold CV	LOOCV	
BPR	0.05	0.61	0.63	0.45 (0.003)	0.36 (0.04)	0.46 (0.003)	0.36 (0.04)	0.41
FPRi	0.24	0.68	0.75	0.57 (0.002)	0.38 (0.03)	0.57 (0.002)	0.38 (0.03)	0.48
FPRe	0.28	0.69	0.73	0.63 (0.002)	0.39 (0.03)	0.64 (0.003)	0.40 (0.04)	0.52
NOP	–	0.32	0.33	0.40 (0.003)	0.08 (0.06)	0.41 (0.003)	0.08 (0.06)	0.24
NOHP	0.22	0.48	0.49	0.51 (0.003)	0.11 (0.08)	0.51 (0.003)	0.12 (0.08)	0.31
MI	0.05	0.39	0.42	0.56 (0.003)	0.16 (0.05)	0.57 (0.002)	0.16 (0.06)	0.36

*Note*: Values into parenthesis () display the standard error of the correlation coefficient estimates.

Abbreviations: BPR, black pod rot; 5‐fold CV, Fivefold cross‐validation; FPRe, frosty pod rot external lesion; FPRi, frosty pod rot internal lesion; LOOCV, leave‐one‐out cross‐validation; NOP, number of pods; NOHP, number of healthy pods; MI, Moniliasis incidence; Trait average: average across all models and cross‐validation designs.

### 3.3 Genomic predictions

The predictive abilities (PAs) for GP models showed promising results across all the traits evaluated (Table 3). Overall, we found no significant differences across models tested (GBLUP_A_ and GBLUP_AD_) for all the traits. However, GBLUP_AD_ (additive + dominance model) showed higher heritability estimates ($h_{\mathrm{AD}}^{2}$). The trait with the highest PA is FPRe, having an average accuracy of 0.52 across models and cross‐validation schemes. In contrast, the trait with the lowest PA is the NOP, with a mean accuracy of 0.24. Predictive abilities for FPR traits (FPRi and FPRe) were higher than BPR and field traits (MI, NOP, and NOHP).

MAS based on significant additive QTLs identified through GWAS exhibited lower predictive abilities than GP across all evaluated traits. MAS predictive abilities ranged from 0.05 to 0.28, whereas GP average predictive abilities ranged from 0.24 to 0.52. Nevertheless, MAS achieved moderate predictive abilities for BPR traits (0.24–0.28).

The TP design had a significant impact on PAs for all traits independent of the GS model (Table 3). We tested two different training designs; the first one was a 5‐Fold CV sampling genotypes across all the families, and the second one was an LOOCV where each family is predicted using a training set that contains genotypes from the remaining families. The PAs were smaller for the LOOCV design comparing 5‐Fold CV, especially for field‐related traits (NOP, NOHP, and MI).

MT‐GP was compared to ST‐GP (GBLUP_A_) in different cross‐validation (CV) schemes (Figure 5). We tested the ability to predict BPR, FPRe, FPRi, and NOP using different combinations of traits which have different degrees of genetic correlations among them (Figure S5). There was a significant increment in the PAs for all traits measured except BPR, when different combinations of traits were used in the MT‐GP models compared to the ST‐GP model. BPR predictive abilities were not improved by adding one or two traits (FPRe and FPRi) in the MT‐GP model, demonstrating no added value of FPR traits in predicting BPR. No extra improvement in PAs was observed between MT‐GP models with two or three traits across all CV schemes and traits evaluated.

**FIGURE 5:**
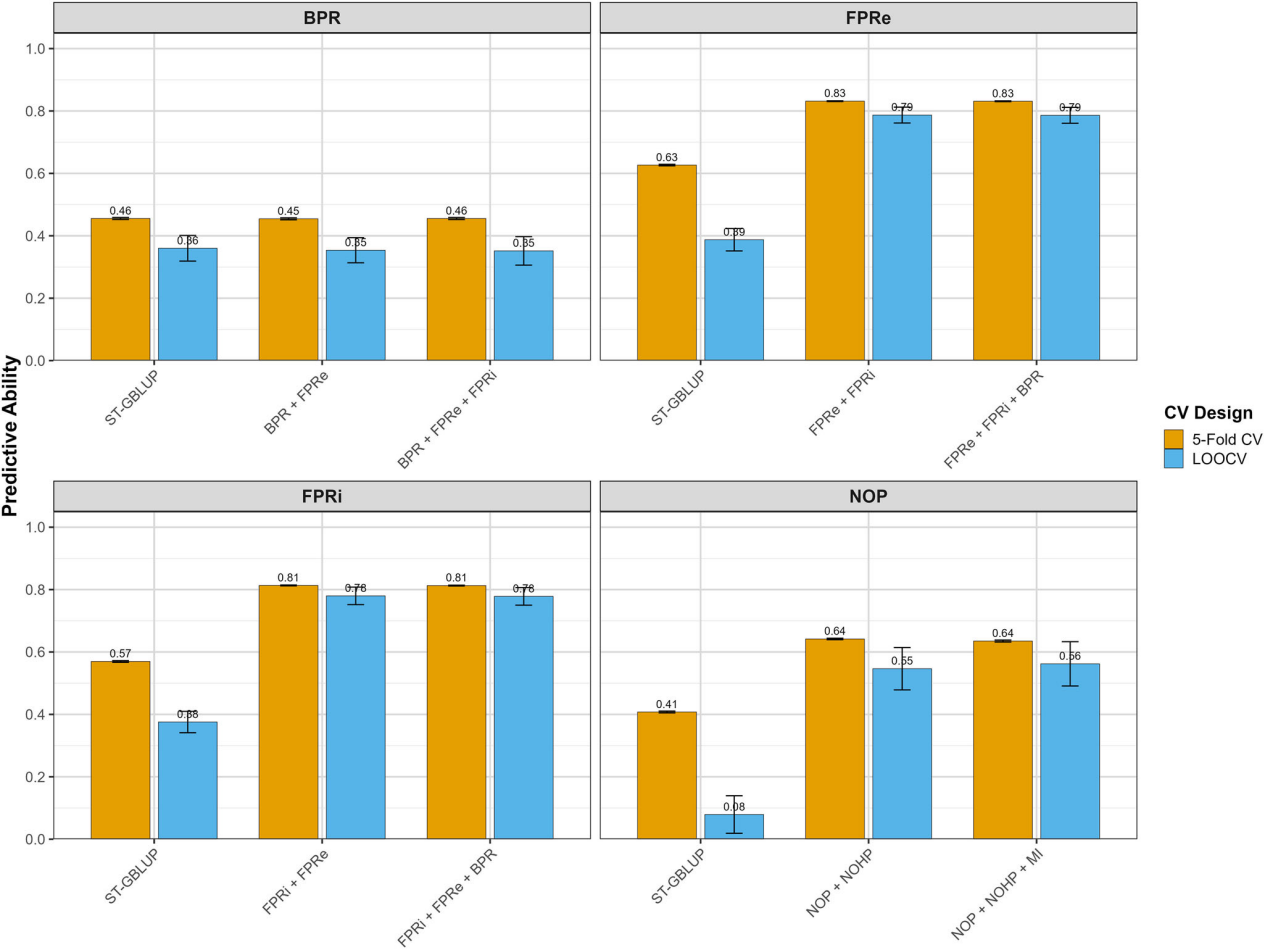
Mean predictive abilities of black pod rot (BPR), frosty pod rot external lesion (FPRe), frosty pod rot internal lesion (FPRi), and number of pods (NOP) for univariate and multivariate genomic GBLUP_A_ prediction models with different combinations of secondary traits. 5‐Fold CV, fivefold cross‐validation; LOOCV, leave‐one‐out cross‐validation; MI, Moniliasis incidence; NOHP, number of healthy pods. The black bars on the top display standard error of the correlation coefficient estimates.

The predictive abilities varied across traits and CV schemes for MT‐GP. For FPRe, the improvements from ST‐GP to MT‐GP were between 1.3‐ and 2‐fold, for FPRi were 1.4‐ and 2‐fold, and for NOP were 1.6‐ and 6.9‐fold. In the ST‐GP model, differences between CV schemes (5‐Fold CV × LOOCV) PAs were significantly bigger; however, in the MT‐GP, PAs performance between CV designs were comparable.

GP is influenced by several factors, one of them in the number of markers, given that we were interested in determining the optimal number of markers that maximize predictive ability without incurring excessive costs with genotyping. We observe a significant gain in predictive ability for all traits up to 1000 markers. Above this number, the gains were minimal (Figure 6), showing a smaller return on predictive ability every time the number of markers was doubled. The trait with the highest predictive ability value was FPRe, achieving an accuracy of 0.61 with 1000 markers and 0.63 with 100,000 markers. A similar trend was observed with the other traits; NOP, a polygenic trait, exhibited the lower predictive ability (0.41) at the full marker density.

**FIGURE 6:**
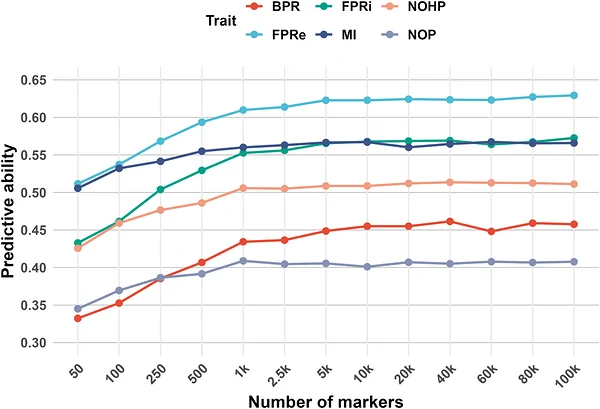
Impact of the number of markers on predictive ability (GBLUP_A_) across six traits. Black pod rot (BPR), frosty pod rot external lesion (FPRe), frosty pod rot internal lesion (FPRi), number of pods (NOP), number of healthy pods (NOHP), and Moniliasis incidence (MI).

Another factor that influences GP is the size of the TP. Our results showed a consistent increase in predictive ability for all traits when adding more individuals to the TP (Figure 7). The largest increases in predictive ability happened between 20 and 200 individuals; FPRe increased from 0.41 (Np = 20) to 0.57 (Np = 200), an increase of 40%, and BPR increased from 0.20 (Np = 20) to 0.38 (Np = 200), an increase of 90%.

**FIGURE 7:**
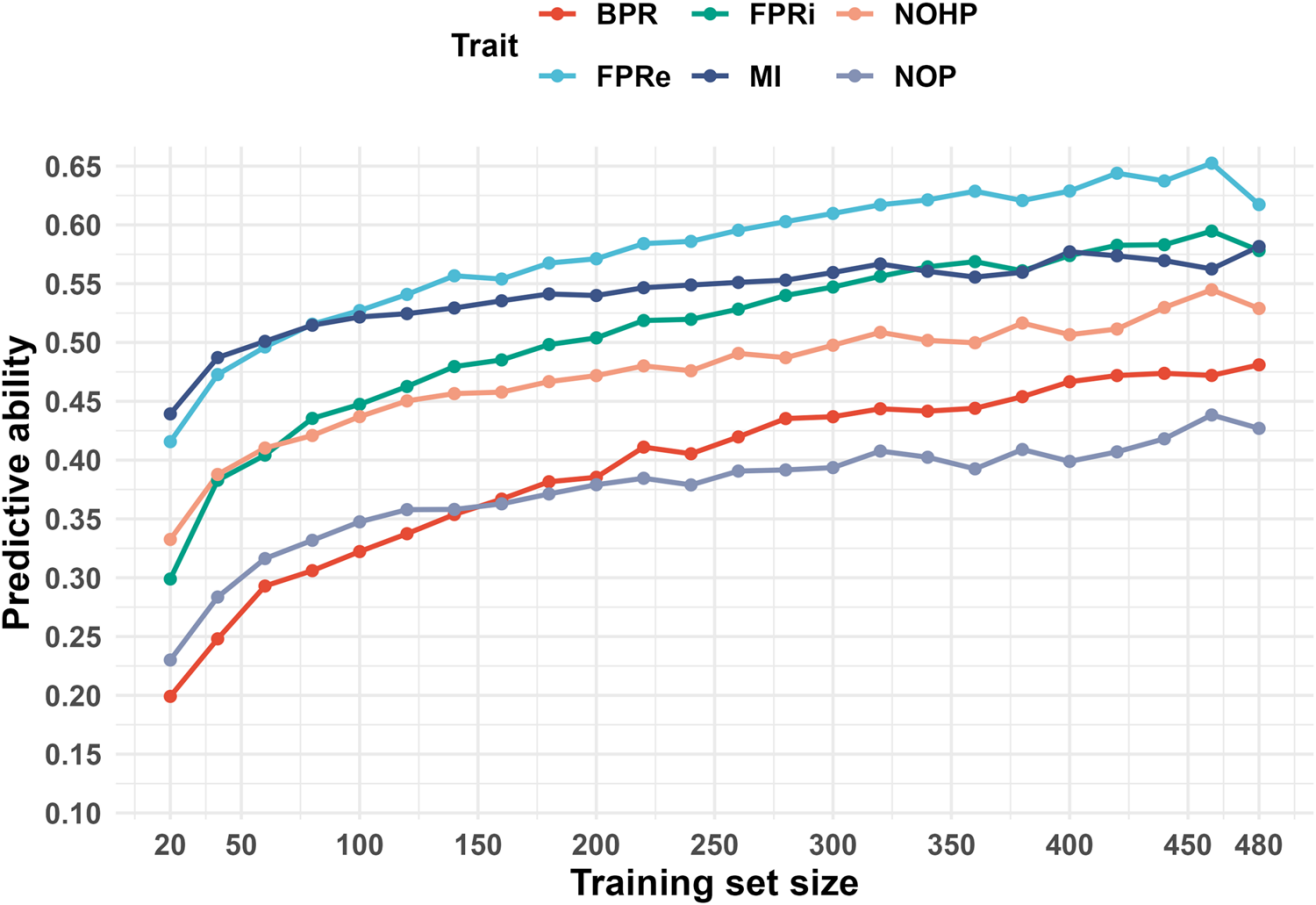
Effect of training population size on predictive ability (genomic best linear unbiased predictor [GBLUP]) across six traits. black pod rot (BPR), frosty pod rot external lesion (FPRe), frosty pod rot internal lesion (FPRi), number of pods (NOP), number of healthy pods (NOHP), and Moniliasis incidence (MI).

## 4 DISCUSSION

This study provides a comprehensive genomic dissection of cacao resistance to FPR and BPR, two major constraints to global cacao production. By integrating GWAS, genomic prediction, and multivariate modeling in a structured multi‐parent population, we demonstrate that resistance to both diseases is highly polygenic, with numerous loci of small effect distributed across all 10 cacao chromosomes. For FPR, 10 regions were associated with FPRi and 21 regions were associated with FPRe. Genomic prediction analyses demonstrated promising predictive abilities for disease‐related traits, ranging from 0.36 to 0.64. These findings reinforce previous reports describing quantitative inheritance of BPR and FPR resistance and highlight the need for breeding strategies capable of capturing genome‐wide variation rather than relying on a limited number of major loci.

### 4.1 FPR and BPR resistance have a complex genetic architecture

Across additive and dominance models, GWAS analysis identified 45 QTLs associated with diseases traits, including a robust hotspot on chromosome 7 consistently associated with BPR, FPRi, and FPRe severity. The colocalization of signals for both diseases in chromosome 7 suggests either pleiotropic defenses or tightly linked resistance mechanisms. The significant associated markers on chromosome 7 do not overlap with previously reported regions in other QTL studies (Brown et al., 2007; Lanaud et al., 2009) or association studies (McElroy et al., 2018; Romero Navarro et al., 2017).

We have identified in our study common QTLs for FPRe and FPRi symptoms on chromosomes 1, 2, 6, 7, and 10, demonstrating the relationship between those two traits and the resistance mechanism. Similar results were observed by Gutiérrez et al. (2021) whom identified regions on chromosomes 4, 7, and 8 associated with FPR internal and external symptoms. Our results were reinforced by the strong genetic correlation identified between FPRi and FPRe scores (0.98, SE = 0.007). Severe internal lesions of FPR can cause the loss of entire beans regardless of the pod's age (Phillips‐Mora et al., 1996). In contrast, external lesions enable *Monilia roreri* mycelium to develop and spread to other trees (Phillips‐Mora et al., 1996). As a result, breeding cacao trees for both symptoms would be the best strategy, since FPR internal resistance has a direct impact on both bean quality and yield, and FPR external resistance could decrease or slow down the FPR spread in the field. However, since both traits are highly genetically correlated, selecting FPRe resistance would also mean selecting FPRi resistance.

QTLs associated with diseases resistance and yield traits have a percentage of the phenotypic variance ranging from 0% to 8%. These results demonstrate a quantitative response, as their genetic variation is governed by the combined effect of several loci, each being responsible for a small proportion of the variation (Semagn et al., 2010). BPR resistance has been characterized as a quantitative trait (Tan & Tan, 1990) with additive and nonadditive components. Crouzillat et al. (2000) demonstrated that there is an epistatic component at least 5 days after inoculation, and Gutiérrez et al. (2021) observed dominance effects in some crosses. Phillips‐Mora et al. (2017) described FPR resistance as polygenic and mostly additive.

Our results demonstrate that BPR and FPR are largely genetically independent traits, a finding supported by the low genetic correlations observed between BPR and both FPRi and FPRe. We also identified a substantial proportion of the phenotypic variance attributable to additive genetic effects across the traits evaluated. However, several disease‐related traits (FPRi, FPRe, BPR, and MI), as well as NOP and NOHP, exhibited genomic regions with significant dominance effects (Table 2). Furthermore, a considerable proportion of phenotypic variation is explained by the dominant effects, attributing higher importance to nonadditive effects on the traits. The genomic heritability for the additive + dominance model ($h_{\mathrm{AD}}^{2}$) shows superior estimates than the additive model ($h_{A}^{2}$) demonstrating the usefulness of dominance in increasing heritability and understanding the genetic mechanism behind the traits. These findings suggest that nonadditive genetic effects could be effectively exploited in clonal breeding programs and in the development of F_1_ hybrids to enhance genetic gain for productivity and disease resistance traits.

### 4.2 Genomic prediction

Genomic prediction results demonstrated promising predictive abilities, ranging from 0.36 for BPR to 0.64 for FPRe. The number of pods, a polygenic trait, displayed the lowest prediction accuracy of 0.08 in LOOCV design. As expected, all prediction accuracies are lower than heritability estimates. Regarding FPR, genomic prediction studies used the number of infected pods by *Monilia roreri* to measure the disease, observed predictive abilities ranging from 0.24 to 0.67 (McElroy et al., 2018; Romero Navarro et al., 2017).

Our prediction accuracies are similar to those reported by Romero Navarro et al. (2017), who observed accuracies of 0.48 for the number of healthy pods, 0.54 for the number of pods infected by frosty pod rot, and 0.67 for the proportion of pods infected by frosty pod rot. A key difference is in how traits were scored: Romero Navarro et al. (2017) relied on counts of healthy or infected pods, whereas we measured lesion severity on a scale separating external and internal lesions. The methodology of disease scoring used in this study displays a more detailed method of phenotyping, as it is possible to classify the resistance levels to FPR internally and externally on a scale between 1 and 5 (see Section 2). Across all six traits, accuracy was similar between genomic prediction models (GBLUP_A_ and GBLUP_AD_), which agreed with accuracies results when comparing models and genetic mode of action (Beche et al., 2021; Colantonio et al., 2022; Xavier et al., 2016).

Although genomic prediction achieved higher predictive abilities than MAS across all traits, the MAS results are nevertheless encouraging. The comparison between the two approaches should be interpreted with caution, as MAS relies on only a limited number of significant markers, whereas genomic prediction captures information from thousands of markers distributed across the genome. Importantly, the primary objective of MAS is not to predict overall genetic performance, but rather to facilitate selection based on a small number of validated loci with favorable effects. Therefore, the predictive abilities obtained with MAS demonstrate that these markers have practical value for breeding applications. However, it is important to emphasize that the identified QTLs require further validation in independent populations and environments before they can be confidently implemented in marker‐assisted breeding programs.

Despite its potential, there are still significant barriers to the global deployment of genomic prediction in cacao breeding. Implementing genomic prediction requires an investment in genotyping capabilities, which must be scaled to thousands of individuals for proper training of the prediction models. Genotyping costs have been decreasing exponentially in the last years, but it is still a challenge for breeding operations with budget constraints. Additionally and importantly, there is a current lack of extensive phenotypic data for model training originating from multi‐location trials. However, the urgent need to increase genetic gain in cacao breeding requires the use of modern breeding techniques. Lubanga et al. (2023) simulated gains of 1.7 times higher for genomic prediction compared to phenotypic selection in a tea breeding program in the seedling stage, reinforcing the value of genomic prediction over other selection methods

### 4.3 Marker density, training population size, and design

Regarding the effective number of markers and TP size, our results suggested that TP size had a greater effect on predictive abilities than the number of markers. Predictive abilities had consistent gains up to 450 individuals. Relatedness of individuals in the TP has more impact on model accuracy than the size of the training set alone (Grattapaglia, 2022). Since our TP is composed of populations interconnected by half‐sibs, it is expected to have higher genetic relatedness between individuals, which contributes to higher predictive ability results. Having an optimal number of markers is crucial for achieving high predictive abilities while maintaining a cost‐efficient genomic prediction strategy. In this study, predictive ability was evaluated using random subsets of SNP markers. Predictive ability increased up to approximately 1000 SNPs, beyond which only marginal gains were observed, indicating a plateau. Similar results were identified by Romero Navarro et al. (2017) in cacao. Importantly, reducing the number of SNPs required for genomic prediction can substantially decrease genotyping costs by enabling the development and use of lower‐density, more affordable genotyping platforms. The relatively low number of markers needed to achieve high predictive abilities is likely due to the high levels of LD observed in the population (Figure S7) and the limited number of recombination events generated through recent breeding cycles. Similar trends have been reported in other perennial species, where predictive ability showed only small improvements beyond 10,000 markers (Grattapaglia, 2022). However, simulations have demonstrated that higher marker densities may maintain genomic prediction accuracy over more breeding generations and potentially result in greater long‐term genetic gains (Long et al., 2011).

It is important to note that the apparent optimum of approximately 1000 SNPs identified in this study is specific to the population evaluated, which consisted of eight biparental families. Therefore, this marker density may not be sufficient for larger and more genetically diverse breeding populations encompassing many unrelated or distantly related families. In such populations, a greater number of markers may be required to adequately capture genetic relationships and linkage disequilibrium patterns, thereby maintaining predictive accuracy across the broader breeding germplasm

The TP design also plays an important role in the GP. It is well known that higher genomic relationships between individuals/populations increase PAs and selection accuracy. We evaluated two cross‐validation scenarios: (i) 5‐fold CV, in which individuals from all families were represented in both the training and validation sets; and (ii) LOOCV, where each family was predicted using a TP composed exclusively of the remaining families. PAs obtained under LOOCV were consistently lower than those observed in the 5‐fold CV scenario, highlighting the importance of family representation and relatedness between the training and validation populations for genomic prediction accuracy.

This validation strategy closely reflects real breeding conditions, in which newly developed populations are predicted using historical TPs that share different levels of genetic relatedness with the target population. The reduced predictive ability under LOOCV indicates that prediction accuracy depends strongly on the relationship between training and validation populations, emphasizing the difficulty of predicting completely untested families in breeding programs.

### 4.4 Secondary traits improve predictive abilities

The higher PAs observed in the MT‐GP models for NOP, FPRi, and FPRe suggest the possibility of exploring multi‐trait to increase accuracy. Particularly, for low‐heritability traits, such as NOP, which present low accuracy under the GBLUP model, the use of secondary traits with higher heritability and correlated to the primary trait increases the PA of the models. Similar results were identified by Dhakal et al. (2025) using grain quality traits in MT‐GP models, with which they observed improvements between 21% and 44% for eating traits and primary cooking in rice. Besides the heritability of the secondary trait and the correlation between the primary and secondary traits, other factors such as complexity of the primary trait, statistical model, and relationship between training and testing set also influence the accuracy (Dhakal et al., 2024).

A higher increment in FPR PAs was observed when using MT‐GP models FPRe + FPRi or vice versa. This was particularly due to the high genetic correlation between FPRe and FPRi (0.98, SE = 0.007) and high heritability of the traits. In contrast, no increment in PAs was observed for BPR when comparing the ST‐GBLUP model and the BPR + FPRe and BPR + FPRe + FPRi MT‐GP models, probably due to the low genetic correlation between BPR and FPR traits. Similarly, NOP benefits from using NOHP in an MT‐GP model, with a significant improvement in the PAs. The incorporation of secondary traits shows great potential for increasing accuracies for primary traits. However, they should not be a burden to phenotype, since this will increase the cost associated with trials and make multivariate GP models a challenge to apply in real conditions.

The limited improvement observed when more than one additional secondary trait was incorporated into the MT‐GP model suggests that not all secondary traits have useful information for predicting the primary trait. The benefit of MT‐GP relies primarily on the existence of substantial genetic correlations among traits, allowing information to be borrowed across traits. When secondary traits exhibit weak genetic correlations with the target trait, they provide little additional information and consequently result in limited gains in predictive ability (Jia & Jannink, 2012). Furthermore, secondary traits that are highly correlated with each other may contain largely redundant information, such that adding multiple secondary traits does not contribute substantially beyond what is already captured by the first correlated trait.

The same training design used previously was tested in an MT‐GP (5‐Fold CV × LOOCV). The PAs differences between CV designs were much larger on the univariate model (GBLUP), compared with the multivariate model (MT‐GP). LOOCV scenario is more challenging to predict, since we have different levels of relationships between families. However, in the MT‐GP, even under limited genetic relationships, the use of secondary traits helped increase prediction accuracy for the primary trait. In the context of a breeding operation, where new crosses/families are generated constantly, and degrees of relationship between families vary among trials and years, secondary traits might be an alternative to achieve higher accuracies of complex traits.

Genomic prediction models have evolved to include multi‐environment models that can better take into account nongenetic variability by integrating agronomic data (Cooper & Messina, 2021). In the same context, crop growth models and environmental data (envirotyping) can be used to improve prediction accuracies (Costa‐Neto et al., 2021). Recently, Hudson et al. (2024) developed host‐by‐pathogen interaction genomic prediction models, which use genomic relationships matrices from the host and pathogen isolates to estimate prediction accuracies. This approach seems promising for systems where host resistance and pathogen virulence are context‐dependent.

## 5 CONCLUSIONS

Our results showed the complex genetic architecture of FPR and BPR resistance in cacao and the potential to use genomic prediction to improve selection accuracy for disease traits. GP models outperform MAS for all the traits evaluated. There was an increase in predictive ability when marker density and TP size increases; marker density plateaued at 1000 markers with little improvements after that, which demonstrates the possibility of exploiting a relatively low marker density for genotyping without decreasing the accuracy of the GP models. The incorporation of secondary traits into a multi‐trait analyses shows great potential for increasing accuracies for primary traits, especially for FPRi, FPRe, and NOP, mainly due to the higher correlation between primary and secondary traits and higher heritability for the secondary traits. The incorporation of genomic selection in cacao breeding, not only for disease resistance but for quality, climatic‐resilient, and yield‐related traits, presents an enormous potential to accelerate genetic gain, especially in a species such as cacao with a longer breeding cycle (3–5 years).

## AUTHOR CONTRIBUTIONS

**Elisa Jacquotte**: Data curation; formal analysis; methodology; visualization; writing—original draft. **James A. Holzwarth**: Data curation; formal analysis; software; writing—review and editing. **Ana Zotta Mota**: Data curation; formal analysis; software; writing—review and editing. **Mariela E. Leandro‐Muñoz**: Investigation; methodology. **Bénédicte Rhoné**: Investigation; methodology. **Evelyn T. Todd**: Formal analysis; software. **Maud Lepelley**: Writing—review and editing. **Laurence Bellanger**: Investigation; methodology. **Patrick Descombes**: Resources; writing—review and editing. **Fabrizio Arigoni**: Resources; writing—review and editing. **Audrey Fillodeau**: Conceptualization; project administration; writing—review and editing. **Eduardo Beche**: Conceptualization; formal analysis; project administration; supervision; writing—original draft.

## CONFLICT OF INTEREST STATEMENT

The authors declare no conflicts of interest.

## Supporting information



Supplemental Material
The supplemental materials include images of the diseases scores for frosty pod rot and black pod rot, a summary of statistics for the phenotypic data, correlation matrix for the phenotypic data, correlation matrix for the genetic correlation among traits, QQ plots for the GWAS analysis, and LD plot.

## Data Availability

Data will be available in DRYAD repository: https://doi.org/10.5061/dryad.0rxwdbsg4.
