# Correction to “Association mapping and genome prediction reveal a complex genetic architecture for resistance to black and frosty pod rot in cacao (*Theobroma cacao* L.) populations”

**DOI:** 10.1002/tpg2.70341

**Published:** 2026-11-09

Jacquotte, E., Holzwarth, J. A., Mota, A. Z., Leandro‐Muñoz, M. E., Rhoné, B., Todd, E. T., Lepelley, M., Bellanger, L., Descombes, P., Arigoni, F., Fillodeau, A., & Beche, E. (2026). Association mapping and genome prediction reveal a complex genetic architecture for resistance to black and frosty pod rot in cacao (*Theobroma cacao* L.) populations. *The Plant Genome, 19*, e70322.https://doi.org/10.1002/tpg2.70322

Table 3 is missing column headers for columns 3 and 4.

The original Table appeared as:

**TABLE 3:** Predictive ability of the genomic prediction models (GBLUP_A_ and GBLUP_AD_) across six traits in cacao.

Trait	MAS			GBLUP_A_		GBLUP_AD_		Trait average
				5‐Fold CV	LOOCV	5‐Fold CV	LOOCV	
BPR	0.05	0.61	0.63	0.45 (0.003)	0.36 (0.04)	0.46 (0.003)	0.36 (0.04)	0.41
FPRi	0.24	0.68	0.75	0.57 (0.002)	0.38 (0.03)	0.57 (0.002)	0.38 (0.03)	0.48
FPRe	0.28	0.69	0.73	0.63 (0.002)	0.39 (0.03)	0.64 (0.003)	0.40 (0.04)	0.52
NOP	–	0.32	0.33	0.40 (0.003)	0.08 (0.06)	0.41 (0.003)	0.08 (0.06)	0.24
NOHP	0.22	0.48	0.49	0.51 (0.003)	0.11 (0.08)	0.51 (0.003)	0.12 (0.08)	0.31
MI	0.05	0.39	0.42	0.56 (0.003)	0.16 (0.05)	0.57 (0.002)	0.16 (0.06)	0.36

*Note*: Values into parenthesis () display the standard error of the correlation coefficient estimates.

Abbreviations: 5‐fold CV, Fivefold cross‐validation; BPR, black pod rot; FPRe, frosty pod rot external lesion; FPRi, frosty pod rot internal lesion; LOOCV, leave‐one‐outcross‐validation; MI, Moniliasis incidence; NOHP, number of healthy pods; NOP, number of pods; Trait average: average across all models and cross‐validation designs.

The updated Table 3 is shown below:

**Table:** 

Trait	MAS			GBLUP_A_		GBLUP_AD_		Trait average
		$h_{A}^{2}$	$h_{\mathrm{AD}}^{2}$	5‐Fold CV	LOOCV	5‐Fold CV	LOOCV	
BPR	0.05	0.61	0.63	0.45 (0.003)	0.36 (0.04)	0.46 (0.003)	0.36 (0.04)	0.41
FPRi	0.24	0.68	0.75	0.57 (0.002)	0.38 (0.03)	0.57 (0.002)	0.38 (0.03)	0.48
FPRe	0.28	0.69	0.73	0.63 (0.002)	0.39 (0.03)	0.64 (0.003)	0.40 (0.04)	0.52
NOP	–	0.32	0.33	0.40 (0.003)	0.08 (0.06)	0.41 (0.003)	0.08 (0.06)	0.24
NOHP	0.22	0.48	0.49	0.51 (0.003)	0.11 (0.08)	0.51 (0.003)	0.12 (0.08)	0.31
MI	0.05	0.39	0.42	0.56 (0.003)	0.16 (0.05)	0.57 (0.002)	0.16 (0.06)	0.36

We apologize for this error.

