# Supplementary material for: Association mapping and genome prediction reveal a complex genetic architecture for resistance to black and frosty pod rot in cacao (Theobroma cacao L.) populations
Source: Plant Genome. 2026 Oct 11;19(4):e70322. doi: 10.1002/tpg2.70322 (PMC13655221; doi:10.1002/tpg2.70322)
Supplement: Supplementary file 1 [file TPG2-19-e70322-s001.docx]

**Supplemtal Tables and Figures**

Association mapping and genome prediction reveals a complex genetic architecture for resistance to black and frosty pod rot in cacao (*Theobroma cacao* L.) populations

1 Elisa Jacquotte - Société des Produits Nestlé SA, Nestlé Research, Tours, France.

2 James A. Holzwarth - Société des Produits Nestlé SA, Nestlé Research, Lausanne, Switzerland.

3 Ana Zotta Mota - Société des Produits Nestlé SA, Nestlé Research, Tours, France.

4 Mariela E. Leandro-Muñoz - CATIE (Centro Agronómico Tropical de Investigacion y Enseñanza) Turrialba, Cartago, Costa Rica.

5 Bénédicte Rhoné - CIRAD, UMR AGAP Institut, F-34398 Montpellier, France

AGAP Institut, Univ Montpellier, CIRAD, INRAE, Institut Agro, Montpellier, France

CATIE (Centro Agronómico Tropical de Investigacion y Enseñanza) Turrialba, Cartago, Costa Rica.

6 Evelyn T. Todd - Société des Produits Nestlé SA, Nestlé Research, Tours, France; present address: Center for Evolutionary Hologenomics, The GLOBE Institute, University of Copenhagen, Copenhagen, Denmark.

7 Maud Lepelley - Société des Produits Nestlé SA, Nestlé Research, Tours, France.

8 Laurence Bellanger - Société des Produits Nestlé SA, Nestlé Research, Tours, France.

9 Patrick Descombes - Société des Produits Nestlé SA, Nestlé Research, Lausanne, Switzerland.

10 Fabrizio Arigoni - Société des Produits Nestlé SA, Nestlé Research, Lausanne, Switzerland

11 Audrey Fillodeau - Société des Produits Nestlé SA, Nestlé Research, Tours, France.

12 Eduardo Beche - Société des Produits Nestlé SA, Nestlé Research, Tours, France.

*Corresponding author: eduardo.beche@rd.nestle.com

**Supplemental Figure S1.** Lesion diameter-based rating scale used to assess resistance to Black pod rot (BPR) - *Phytophthora palmivora.*

**Supplemental Figure S2.** Rating scale to assess resistance to Frost pod rot external (FPRe) - *Moniliophthora roreri*.

**Supplemental Figure S3.** Rating scale to assess resistance to Frost pod rot internal (FPRi) - *Moniliophthora roreri*.

**Supplemental Table S1.** Summary of statistics for black por rot (BPR), frosty pod rot internal (FPRi), frosty pod rot external (FPRe), number of pods (NOP), number of healthy pods (NOHP), and Moniliasis incidence (MI) for eight populations in cacao.

| **Cross** |  | **Trait** | | | | | |
| --- | --- | --- | --- | --- | --- | --- | --- |
|  |  | **BPR** | **FPRi** | **FPRe** | **NOP** | **NOHP** | **MI** |
|  |  | **(cm)** | **(1-5)** | **(1-5)** |  |  | **(%)** |
| CATIE-R6  x  CHUAO-120 | Mean | 22 | 2.5 | 2.2 | 13 | 11 | 11.2 |
|  | SD | 7 | 0.9 | 0.7 | 7.4 | 6.4 | 8.8 |
|  | Range | 2-30 | 1.2-5 | 1.2-5 | 1.2-31 | 1.2-28.5 | 0-48.6 |
|  | CV (%) | 32.3 | 35.2 | 33.1 | 57.2 | 57.2 | 78.9 |
| CATIE-R6  x  EET-95 | Mean | 24 | 3 | 2.4 | 11 | 9 | 14.4 |
|  | SD | 6.3 | 1 | 0.7 | 6.6 | 6.1 | 13.6 |
|  | Range | 4.2-30 | 1-5 | 1-4.5 | 1.5-32.5 | 1-32 | 0-66.1 |
|  | CV (%) | 25.7 | 33 | 28.5 | 60.8 | 65.6 | 94.5 |
| CATIE-R6  x  PA-51 | Mean | 19 | 2.4 | 1.9 | 7 | 6 | 7 |
|  | SD | 8.3 | 1.2 | 0.7 | 3.5 | 3.2 | 8.9 |
|  | Range | 2-30 | 1-5 | 1-3.6 | 1.25-16.2 | 1-15 | 0-37.5 |
|  | CV (%) | 43.2 | 49.5 | 37.3 | 50.2 | 49.9 | 126 |
| CHUAO-120  x  ICS-95 T1 | Mean | 26 | 3.1 | 2.8 | 8 | 5 | 32.3 |
|  | SD | 6 | 1.2 | 0.9 | 5.1 | 3.4 | 18 |
|  | Range | 4.5-30 | 1.5-5 | 1.5-4.67 | 1.25-20 | 1-14.8 | 2.78-83.9 |
|  | CV (%) | 22.9 | 37.1 | 32.6 | 62.8 | 68.6 | 55.9 |
| EET-399  x  CATIE-R6 | Mean | 25 | 3 | 2.3 | 14 | 12 | 12.4 |
|  | SD | 5 | 0.9 | 0.6 | 6.3 | 6 | 10.3 |
|  | Range | 6-30 | 1.2-5 | 1.2-3.8 | 2.2-36.2 | 1.2-33.5 | 0-53.3 |
|  | CV (%) | 20.5 | 30.2 | 24.5 | 44.6 | 48.4 | 83.3 |
| EET-399  x  ICS-95 T1 | Mean | 23 | 3.7 | 3.1 | 11 | 7 | 28.6 |
|  | SD | 7.2 | 1.1 | 1 | 7 | 4.3 | 19.3 |
|  | Range | 2-30.4 | 1.4-5 | 1.3-5 | 1.5-32.5 | 0.75-20.5 | 0-86.2 |
|  | CV (%) | 31.8 | 29.3 | 31.5 | 63.1 | 62.8 | 67.4 |
| EET-95  x  ICS-95 T1 | Mean | 26 | 3.9 | 3.5 | 9 | 5 | 37.2 |
|  | SD | 5.6 | 1.1 | 1.1 | 5.3 | 3.9 | 21.3 |
|  | Range | 3.3-30 | 1.8-5 | 1.5-5 | 1.25-26 | 0.5-20.5 | 0-75.8 |
|  | CV (%) | 21.7 | 28.2 | 31 | 57.7 | 73.2 | 57.2 |
| PA-51  x  ICS-95 T1 | Mean | 26 | 3.7 | 3.3 | 6 | 4 | 22.3 |
|  | SD | 8.4 | 1.1 | 1.1 | 3 | 2.5 | 17.2 |
|  | Range | 2-30 | 1.38-5 | 1.25-5 | 1-11.8 | 0.5-10.8 | 0-72.2 |
|  | CV (%) | 43.6 | 30.4 | 32.6 | 52.7 | 58.4 | 76.9 |

SD, Standard deviation; CV(%), Coefficient of variation.

**Supplemental Figure S4.** Spearman’s phenotypic correlation coefficients for black por rot (BPR), frosty pod rot internal (FPRi), frosty pod rot external (FPRe), number of pods (NOP), number of healthy pods (NOHP), and Moniliasis incidence (MI) for eight populations in cacao. Correlation is significantly different from zero when P-value < 0.05. A cross sign marks non-significant correlations.

**Supplemental Figure S5.** Correlation matrix of genetic correlations estimated by a multivariate model (lower diagonal) and their respective standard error (SE - higher diagonal). The matrix display all the combinations between black pod rot (BPR), frosty pod rot internal (FPRi), frosty pod rot external (FPRe), number of pods (NOP), number of healthy pods (NOHP), and Moniliasis incidence (MI) for eight populations in cacao.

**Supplemental Figure S6.** QQ Plots for six traits of cacao under the LOCO Model for additive and dominance: Black pod rot (BPR), Frosty pod rot internal (FPRi), Frosty pod rot external (FPRe), Number of pods (NOP), Number of healthy pods (NOHP), and *Moniliasis* incidence (MI).

**Supplemental Figure S7.** Linkage disequilibrium (LD) decay in the cacao panel.
